# T Cell Receptor Profiling in Type 1 Diabetes

**DOI:** 10.1007/s11892-017-0946-4

**Published:** 2017-10-11

**Authors:** Laura M. Jacobsen, Amanda Posgai, Howard R. Seay, Michael J. Haller, Todd M. Brusko

**Affiliations:** 1 Department of Pediatrics, College of Medicine, University of Florida Diabetes Institute, Gainesville, FL USA; 2 Department of Pathology, Immunology and Laboratory Medicine, College of Medicine, University of Florida Diabetes Institute, Gainesville, FL USA

**Keywords:** T cell receptor, Immunosequencing, Type 1 diabetes, Human immune repertoire, Adaptive immunity, Autoimmunity

## Abstract

**Purpose of Review:**

The genetic susceptibility and dominant protection for type 1 diabetes (T1D) associated with human leukocyte antigen (HLA) haplotypes, along with minor risk variants, have long been thought to shape the T cell receptor (TCR) repertoire and eventual phenotype of autoreactive T cells that mediate β-cell destruction. While autoantibodies provide robust markers of disease progression, early studies tracking autoreactive T cells largely failed to achieve clinical utility.

**Recent Findings:**

Advances in acquisition of pancreata and islets from T1D organ donors have facilitated studies of T cells isolated from the target tissues. Immunosequencing of TCR α/β-chain complementarity determining regions, along with transcriptional profiling, offers the potential to transform biomarker discovery.

**Summary:**

Herein, we review recent studies characterizing the autoreactive TCR signature in T1D, emerging technologies, and the challenges and opportunities associated with tracking TCR molecular profiles during the natural history of T1D.

## Introduction

Abundant evidence in animal models support the central role T cells play in the adaptive immune-mediated killing of pancreatic β-cells in type 1 diabetes (T1D) [1–4]. This direct pathogenic role is corroborated by bone marrow and adoptive cell transfers in both non-obese diabetic (NOD) mice and patients [5–8], along with T cell receptor (TCR) transgenic models [9]. Yet, key differences have been noted in the prominent lymphocytic infiltrate present in the NOD mouse in comparison to the variable and often sparse insulitis observed in the pancreas of organ donors with T1D [10, 11]. These differences have prompted extensive efforts to further investigate disease mechanisms and the nature of the autoreactive response in human beings [10–14]. The capacity to acquire tissues directly from pancreas and islets of organ donors with T1D has provided a unique opportunity to study tissue-resident lymphocytes and elucidate their phenotype and molecular signature.

The search for biomarkers of T cell autoreactivity in T1D has largely been driven by efforts to identify T cell peptide epitopes derived from antigens recognized by autoantibodies [15, 16]. These studies have employed MHC-multimers, ELISpot, and T cell dye dilution assays, among others [17–19]. A significant number of clones and TCR reactivities have emerged; however, the adoption of bioassays to track autoreactive T cells has largely remained restricted to individual investigator laboratories. The lack of widespread adoption of such assays likely results from both biological and technical limitations [i.e., requirements for significant numbers of viable peripheral blood mononuclear cells (PBMC) and assays based on specific human leukocyte antigen (HLA) haplotypes]. Moreover, the antigen specificity of the TCR is thought to direct and retain T cells within defined tissues and draining lymph nodes (LN) [20–22], making the circulating frequency of autoreactive T cells exceptionally low—estimated at less than 0.05% CD4^+^ T cells [23•] and 0.01% of CD8^+^ T cells [24, 25]. This low frequency restricts the numbers of antigens and epitopes available for analysis and requires considerable volumes for current assays, a notable limitation in pediatric patients. To address some of these limitations and further explore the potential of TCR repertoire analysis, we along with others have begun to employ immunosequencing of TCRs to identify T cell biomarkers with a high level of sensitivity and specificity. With this review, we summarize current progress in the field of T cell sequencing technologies, the potential for novel biomarker discovery, and the application of this knowledge base toward developing new therapies to cure T cell-mediated autoimmune diseases, with a specific focus on T1D.

## Role for Autoreactive T Cells in T1D Pathogenesis

### Dominance of HLA Risk

The HLA-DR3/DR4-DQ8 haplotype carries the highest risk for progression to T1D [26]. HLA molecules shape the developing TCR repertoire during thymic selection. Developing T cells integrate signals from recombination events resulting in fate determinations to the TCR γ/δ or α/β lineage. Among the α/β TCR lineage, positive and negative selection events further shape the repertoire including the emergence of thymic-derived regulatory T cells (tTreg), which are necessary to establish peripheral immune tolerance [27]. Approximately 57 genetic loci identified by genome-wide association studies (GWAS) (e.g., *PTPN22*, *CD25*, *SH2B3*, *IFIH1*, *CD226*, etc.), each with low individual odds ratios, are thought to provide additional risk besides HLA, by promoting innate inflammation, leading to altered immune cell signaling, and augmenting β-cell stress, ultimately resulting in a failure in immune tolerance [28]. Importantly, HLA haplotypes and other susceptibility alleles are carried at varying frequencies in different ethnic groups. Even among groups with similar background genetics, geographic exposures may result in highly variable disease penetrance. Combinations of alleles can also provide dominant protection, as noted for DQB1*06:02 in Caucasians as well as DPB1 polymorphisms in non-Caucasian ethnicities [29, 30]. Moving forward, it will become important to understand both the impact of allelic frequencies and the role of environmental exposures in determining the diversity and specificity of the TCR repertoire in various T1D cohorts.

### T Cell Studies in nPOD and Following Pancreatic Transplant

Over the past decade, the Network for Pancreatic Organ donors with Diabetes (nPOD) program has facilitated investigations of the T1D pancreas across a wide range of donor ages, races, and T1D disease durations [31]. The insulitis lesion that histologically characterizes T1D is much less fulminant in the human pancreas than in the NOD mouse [32, 33]. Human insulitis has been shown to include macrophages, natural killer (NK) cells, B cells, and CD3^+^ T cells, with a predominance of CD8^+^ relative to CD4^+^ T cells. A relationship between this CD8^+^ T cell predominance within insulitis and HLA class I hyperexpression in islets has been observed exclusively in individuals with T1D [34]. CD8^+^ T cells are under increased scrutiny to identify key receptors and characterize their functional role in T1D development for potential use as biomarkers or therapeutic targets. Indeed, insulitic CD8^+^ TCR reactivities appear unique to individual islets in the limited number of recent-onset T1D organ donors examined so far; perhaps these represent local clonal expansions, with nearby islets demonstrating autoreactivity to distinct epitopes/autoantigens [34]. Moreover, HLA-A*02-01-tetramer staining for epitopes derived from six known T1D autoantigens demonstrated glucose-6-phosphatase 2 (G6Pase 2)-reactive CD8^+^ T cells to be the most prevalent within lesions examined from 45 T1D donors of varying disease durations [34]. Insulitic lesions were specific for a single β-cell autoantigen in donors with recent-onset disease whereas diverse T cell autoreactivity was observed in the majority of donors with longstanding disease. Lack of autoreactivity to any epitope tested was noted from two long duration (≥ 1 year) cases, even in the presence of insulitis [34]. Monospecificity early in disease followed by oligoclonal T cell expansion may be seen within human islets as T1D advances, similar to epitope spreading demonstrated in the NOD mouse [35]. Efforts to investigate this phenomenon in human tissues are limited by the inability to perform longitudinal studies of the T1D pancreata. Nevertheless, we predict that insulitis lesions in human pancreata with long- versus short-duration T1D will show evidence of similar evolution over time.

Antigen-driven expansion of autoreactive T cells occurs in T1D patients who have undergone pancreas transplant with recurrent T1D. This process is marked by sudden and severe hyperglycemia, the appearance of new islet autoantibodies [i.e., glutamic acid decarboxylase 65 (GAD65), insulinoma-associated protein-2 (IA-2), zinc transporter 8 (ZnT8)], and expansion of autoreactive T cells. Circulating T cell autoreactivity varied across transplant recipients with GAD65-specific CD4^+^ T cells identified from two patients and G6Pase 2-reactive CD8^+^ T cells from a third. In one patient, GAD65-reactive CD4^+^ T cells were present in the peripheral blood, the transplanted pancreas, and pancreas draining LN (pLN) with persistent detection of a single TCR β-chain complementarity determining region 3 (CDR3), despite immunosuppressive treatment [36•]. A fourth transplant recipient had GAD65-specific CD8^+^ T cells exhibiting a CCR7^−^ memory phenotype within the pancreas transplant draining LN. Histologically, B cell and T cell insulitis, β-cell loss, and variable degrees of transplant rejection were seen [36•]. These observations highlight that not only is the autoreactive T cell repertoire pathogenic, but also the memory T cell response persists for many years and may display resistance to drugs capable of suppressing allograft rejection.

## Putatively Known Autoreactive TCRs

### Analyses in Peripheral Blood

T cell clones specific for β-cell antigens (e.g., G6Pase 2, insulin, pre-proinsulin, IA-2, GAD65, ZnT8 [37]) have been isolated from peripheral blood of living patients with T1D but can also be detected from control subjects [18, 23•, 24, 38•, 39–42]. We and others have demonstrated high diversity of the TCR repertoire, even within the islets and pLNs of patients with T1D and autoantibody positive subjects who have elevated risk of progressing to disease [23•, 38•]. Functionally, detailed studies have revealed that peripheral blood T cells from HLA-matched T1D and control subjects produce IFN-γ versus IL-10, respectively, in response to activation with autoantigen [43]. Two GAD-reactive TCR clones, 4.13 and R164, isolated from peripheral blood of patients with T1D were derived from shared TCRα and TCRβ variable (V) gene segments recognizing a common peptide target (i.e., GAD_555–567_). These clones have only minor functional differences in the amino acid sequence of the CDR3 domain but exhibit different binding avidities [44]. In vivo studies of humanized TCR-transgenic mice expressing the lower-avidity 4.13 or high-avidity R164 clone revealed differences in thymic selection and peripheral T cell skewing. 4.13 TCR-transgenic mice exhibited a more tolerogenic repertoire including IL-10 producing T cells whereas R164 TCR-transgenic mice were observed to exclusively contain IFN-γ positive T cells [45]. To further examine the functional implications of TCR avidity in vitro, we used lentiviral transduction of primary human T cells to engineer GAD 4.13 and GAD R164 TCR avatars. Compared to 4.13 Tregs, high-avidity R164 Tregs exhibited greater suppressive capacity over antigen-specific responder T cells activated with GAD_555–567_ peptide or bystander CD8^+^ T cells recognizing a MART-1 peptide (Yeh et al., in press). These studies suggest autoreactive TCR avidity likely influences the functions of both Treg and effector T cell (Teff) populations in T1D pathogenesis. However, these studies cannot directly infer diabetogenicity and have led to investigations of T cells isolated from islet tissues of deceased organ donors with T1D.

### Islet Studies

**Fig. 1:**
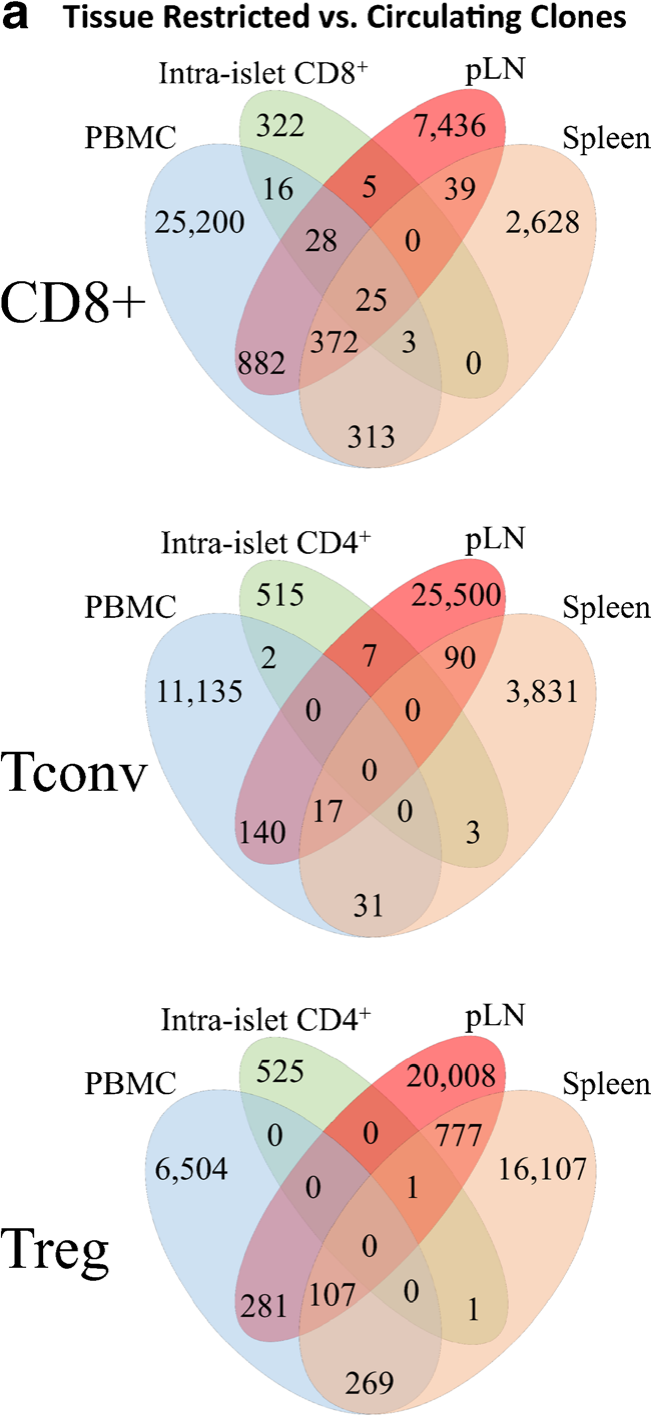

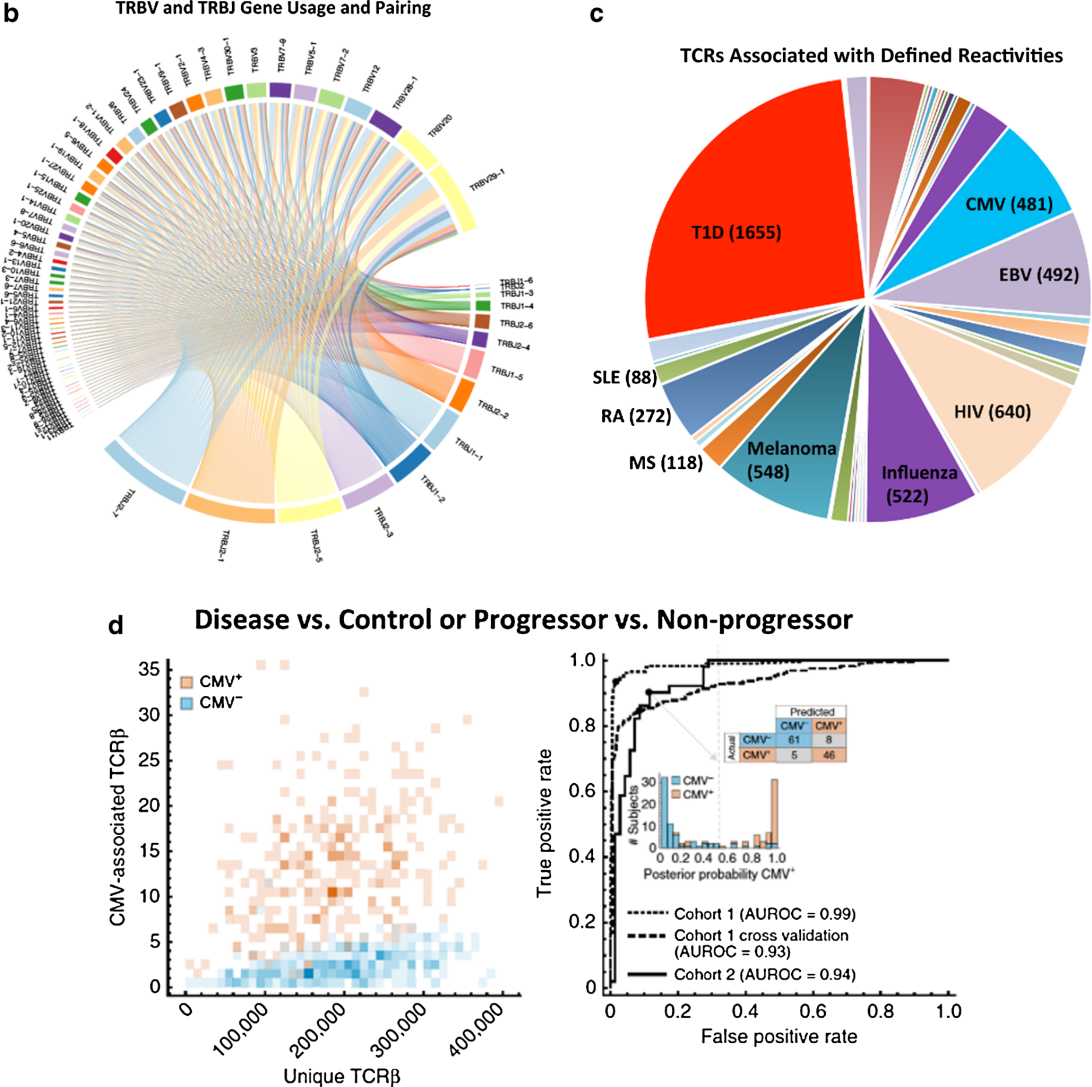
Potential applications for next-generation sequencing (NGS) of the T cell receptor (TCR) repertoire in type 1 diabetes (T1D) research. **a** For the CD8^+^ T cell, CD4^+^ conventional T cell (Tconv), and regulatory T cell (Treg) subsets, the number of TCRβ complementarity determining region 3 (CDR3) amino acid (AA) sequences shared across peripheral blood mononuclear cells (PBMC, blue), intra-islet (green), pancreatic draining lymph node (pLN, red), and spleen (orange) samples from a single donor (Reprinted with permission from: Seay HR, et al. JCI Insight. 2016;1(20):e88242) [38•]. **b** Circos plot depicting *TRBV* and *TRBJ* gene families detected from a single PBMC sample. *Colored ribbons* represent each gene family with frequency corresponding to ribbon width. V-J combinations are indicated by ribbons connecting across the circle. **c** Six thousand three hundred and twenty-one clonotypes of known reactivity are shown here for > 20 diseases with the number of known clonotypes indicated in parentheses for nine of the most abundant: T1D *N* = 1655 (red), human immunodeficiency virus (HIV) *N* = 640 (beige), Influenza *N* = 522 (purple), melanoma *N* = 548 (teal), Epstein-Barr virus (EBV) *N* = 492 (light purple), cytomegalovirus (CMV) *N* = 481 (bright blue), rheumatoid arthritis (RA) *N* = 272 (gray blue), multiple sclerosis (MS) *N* = 118 (orange), and systemic lupus erythematosus (SLE) *N* = 88 (light green) [23•, 46–165]. **d** CMV-associated TCRβ sequences were identified from CMV seropositive (orange) and CMV seronegative (blue) subjects (left). Computer models were trained using CMV-associated TCRβ and CMV seropositivity status data (cohort 1, dashed lines). Models were validated using a separate cohort of individuals (cohort 2, solid line). Receiver operating characteristic (ROC) curve analyses indicate that NGS can identify CMV serostatus with sensitivity = 0.90 and specificity = 0.89; area under ROC (AUROC) = 0.93 for cross-validation of cohort 1 and AUROC = 0.94 for cohort 2 (Reprinted by permission from Macmillan Publishers Ltd.: Emerson RO, et al. Nat Genet. 2017;49(5):659–65) [166••]

In one of the first studies of T cells isolated from a human T1D pancreas, clones from five TCRβ V-gene families (Vβ1, Vβ7, Vβ11, Vβ17, Vβ22) were found to be expanded. One of these clonal expansions, Vβ22, was also expanded in spleen and peripheral blood; however, autoantigen or epitope reactivities were not determined for the expanded clones [46]. We recently reported an extensive analysis of the adaptive repertoire from pLN, spleen, PBMC, and inguinal or mesenteric LN of 18 T1D and nine control organ donors [38•]. These efforts also demonstrated limited clonal overlap between circulation and the target organ (Fig. 1a) and support the notion that studies of peripheral blood T cells alone are likely insufficient to fully characterize the T1D autoimmune repertoire, highlighting the need for studies of intra-pancreatic T cells in T1D.

**Fig. 2:**
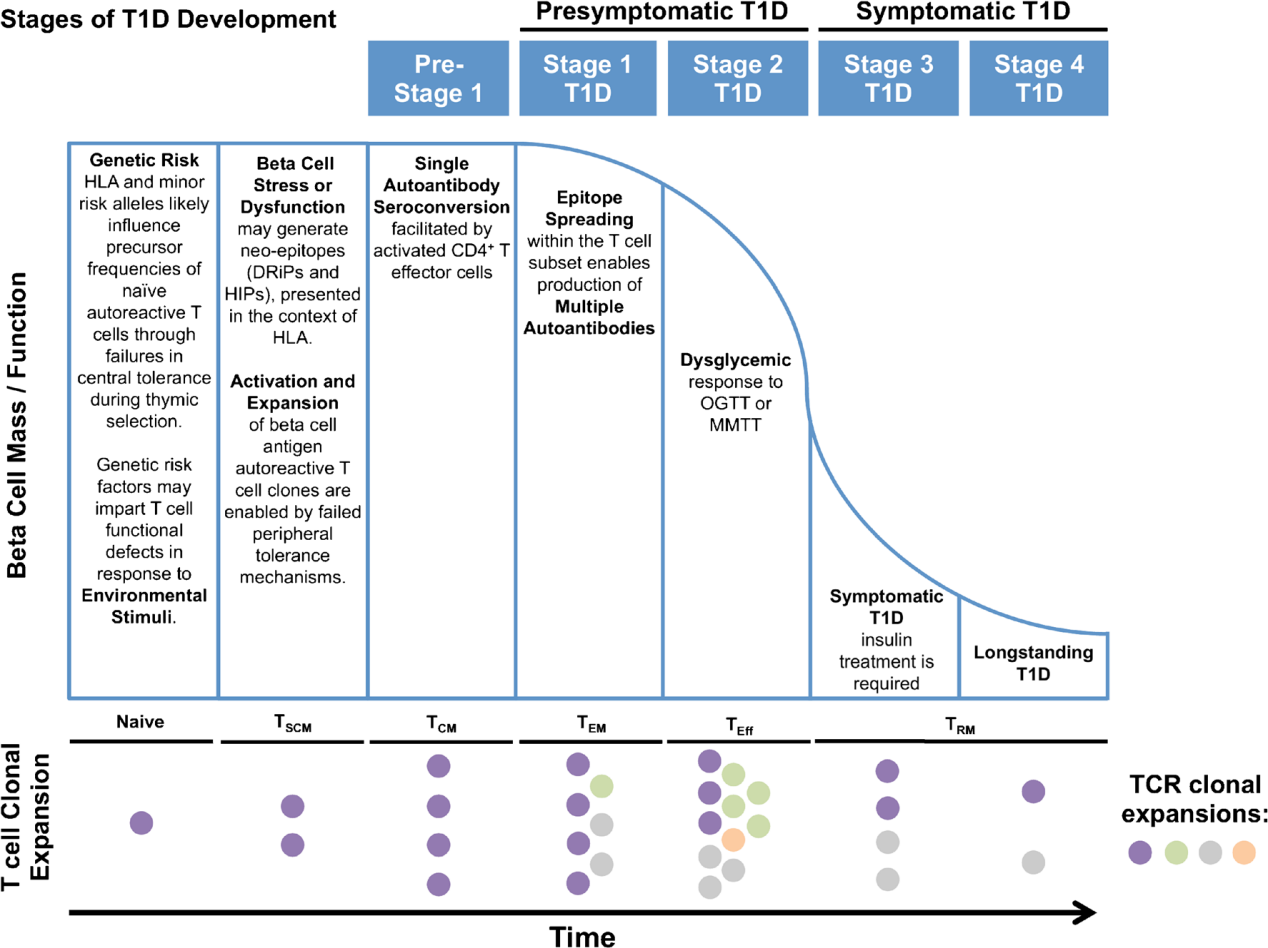
Hypothesized timeline of T cell clonal expansion as it relates to the development of autoantibodies, development of dysglycemia, and loss of β-cell mass and/or function in the natural history of type 1 diabetes (T1D). Staging of T1D is modeled after the 2015 consensus report [170•] and modified from the Eisenbarth model of T1D [171, 172] with pre-disposing factors as well as key immunologic and metabolic events noted over the course of the disease. The T cell expansion diagram reflects development of a naïve T cell clone into stem cell memory T cell (T_SCM_), central memory T cell (Tcm), effector memory T cell (T_EM_), effector T cell (T_Eff_), and tissue resident memory T cell (T_RM_) subsets based on the model presented by Farber et al. [21], with colors (i.e., purple, green, gray, orange) representing T cell receptor (TCR) clonal expansions

As noted above, insulitis is heterogeneous throughout the human T1D pancreas, most often affecting residual insulin-containing islets, and the ability to expand live T cells from isolated human islets containing insulitis represents a remarkable achievement. Indeed, Pathiraja et al. characterized 53 intra-islet CD4^+^ T cell clones and reported that 14/53 reacted with proinsulin [167]. CDR3 sequences were not conserved across the 14 proinsulin-reactive intra-islet T cell clones suggesting that they did not originate from a common parent clone. Similarly, Michels et al. characterized three proinsulin-reactive T cell clones (i.e., 20D11, 6H9, and 8E3) isolated from human T1D islets; 20D11 and EH9 are the first human intra-pancreatic isolates specific for the insulin B:9–23 peptide [168••]. In a recent report by Babon et al., GAD-, proinsulin-, IA-2-, and chromogranin A-reactive CD4^+^ T cell clones were isolated from human islets from organ donors with T1D [169••]. CD8^+^ T cells specific for insulin, IA-2, and G6Pase 2 peptides were also detected. Functionally, antigen-stimulated cytokine secretion varied across clones but, overall, it was pro-inflammatory with a Th1 bias [169••]. Further, our recent study of intra-islet T cells from one T1D donor revealed a TCRβ CDR3 corresponding to the previously identified GAD-reactive clone 4.13; no other known autoreactive clones were identified from the islet sample [38•]. GAD4.13 was identified in LN or spleen samples from six additional T1D donors. These reports highlight the apparent heterogeneity of T1D with regard to T cell antigen reactivity and T cell function/phenotype. These studies also emphasize the need for large datasets to illuminate molecular patterns likely governed by HLA and minor T1D risk alleles (e.g., *INS/IGF2*) that might influence clonal frequency throughout the natural history of disease (Fig. 2).

### T Cells Recognizing Neo-epitopes, Defective Ribosomal Products, and Hybrid Insulin Peptides

Growing evidence supports a role for epitopes derived from defective ribosomal products (DRiPs) and hybrid insulin peptides (HIPs) as potentially initiating autoantigens in T1D (Fig. 2). DRiPs result from dysfunctional or erroneous translation of pre-proinsulin transcripts [173••]. For instance, translation of *INS* mRNA from an alternative open reading frame can generate novel, seemingly aberrant peptides. In a recent report by Kracht et al., CD8^+^ T cells specific for an insulin DRiP peptide were isolated from peripheral blood of patients with T1D and were demonstrated to specifically destroy human islet cells in vitro [173••]. Similarly, HIPs are derived from the post-translational fusion of proinsulin peptides with other β-cell proteins within the secretory granule [174••]. HIP-reactive T cells have been isolated from the pancreata of human organ donors with T1D, and perhaps not surprisingly, the HIP-reactive clones (A2.11 and A3.10) exhibited HLA-DQ8 restriction [174••]. T cells recognizing modified insulin epitopes are likely not subjected to regulation by central tolerance mechanisms, and there is evidence suggesting DRiP and HIP-reactive T cells may be present in both control and T1D subjects [173••]. However, high-risk HLA haplotypes may preferentially present DRiP and HIP neo-epitopes generated within the β-cell, supporting a diabetogenic role. Hence, T cells recognizing DRiPs and HIPs likely represent an important class of potential TCR biomarkers, particularly within the context of certain HLA haplotypes, and may represent candidate targets for novel T cell-directed therapies.

## Technologies for Analyzing TCR Sequences

Being a budding field, high-throughput immunosequencing (REP-seq) does not have a well-defined or standardized methodology for data acquisition and analysis. The reasons for this variability are many but relate to the use of different sample input tissues, sequencing platforms, and data analysis pipelines. The choice of analysis platform is highly dependent upon the needs for sample depth and resolution, along with practical considerations of cost. A number of technologies have been utilized for the acquisition and analysis of TCR sequences, varying from single investigator concepts to turnkey commercial processing pipelines. Below, we review some of the previously utilized approaches and describe emerging technologies for immunosequencing applications (Table 1).

**Table 1:** Technologies for immunosequencing*

Stage in Workflow	Technology	Availability	Input	Single-cell?	TCR/BCR Gene(s)	Platform	Dependencies	Notes	Reference
Experimental Technique	pairSEQ™ (Adaptive Biotechnologies)	Not yet commercially available	Viable cells	No	TRA/TRB-pairing	n/a	n/a	Technique uses diversity of TCR to pair TRA and TRB within an experiment of pooled RNA from multiple cells with the use of the immunoSEQ® pipeline	[52]
TCR Sequencing	SMARTer® TCR Profiling (Takara)	Proprietary Kit	RNA	No	TRA, TRB	n/a	n/a	Kit for NGS of TRA and TRB using pooled RNA from samples ranging from 50 to 10,000 purified T cells	[112, 113]
Pre-processing	VDJPipe	Website, free account	FASTA or FASTQ (DNA)	Yes	TRA, TRB, TRG, TRD, IGL, IGK, IGH	Win, Mac, Linux	Web Browser	Preprocessing of DNA sequence data on VDJserver	[114]
	pRESTO	Open-source	FASTQ (DNA or RNA)	Not recommended	TRA, TRB, TRG, TRD, IGL, IGK, IGH	Win, Mac, Linux	Python v3.4.0 with 8 additional packages	Annotates reads with sequence embedded barcodes and generates unique molecular identifier (UMI) consensus sequences	[115]
	IgBLAST	Public domain	Aligned Data	Yes	TRA, TRB, TRG, TRD, IGL, IGK, IGH	Win, Mac, Linux	BLAST database files, IMGT germline sequences	Identifies germline V(D)J gene segments, details at rearrangement junctions, the delineation of Ig sub-regions	[116, 117]
	MiGMAP	Open-source	Aligned Data	Yes	TRA, TRB, TRG, TRD, IGL, IGK, IGH	Win, Mac, Linux	Java v1.8 and IgBLAST v1.4-1.6.1	A smart wrapper for IgBLAST which includes additional experimental modules for contig assembly, error correction, and immunoglobulin lineage tree construction	[118]
	Decombinator	Open-source	FASTQ files	Yes	TRA, TRB, TRG, TRD	Win, Mac, Linux	Python v2.7 + 4 non-standard modules	Quickly aligns HTS data	[119, 120]
	TraCeR	Open-source	FASTQ (RNA)	Only	TRA, TRB, TRG, TRD	Mac, Linux	Bowtie v2, Trinity, IgBLAST, Kallisto, Graphviz, Oracle Java v1.8, Python v2.7, with 8 additional packages	Reconstruction of T cell receptor sequences from single-cell RNA-seq data	[121, 122]
	MiGEC	Open-source	FASTQ (RNA)	Not recommended	TRA, TRB, TRG, TRD, IGL, IGK, IGH	Mac, Linux	Java and BLAST+	Allows for barcoded molecular identifiers in an attempt to eliminate profiling errors	[123, 124]
	ImSeq	Open-source	FASTA or FASTQ (DNA or RNA)	Not recommended	TRA, TRB, TRG, TRD, IGL, IGK, IGH	Mac, Linux	A C++14 capable compiler and ZLIB	Used to derive clonotype repertoires from NGS data handing errors from PCR and sequencing artefacts	[125, 126]
	RTCR	Open-source	FASTQ (DNA or RNA)	Not recommended	TRA, TRB, TRG, TRD	Mac, Linux	Python v2.7 and an external aligner (e.g. Bowtie 2)	A pipeline for complete and accurate recovery of TCR repertoires from high throughput sequencing data	[127, 128]
	MiXCR	Open-source	FASTQ (DNA or RNA)	Yes	TRA, TRB, TRG, TRD, IGL, IGK, IGH	Win, Mac, Linux	Oracle Java 1.8	Default pipeline can be executed without any additional parameters	[129, 130]
Pre-processing, Data Analysis, and Visualization	VDJserver	Website, free account	FASTA or FASTQ (DNA or RNA)	No	TRA, TRB, TRG, TRD, IGL, IGK, IGH	Win, Mac, Linux	Web Browser	For performing Rep-Seq analysis, as well as storing and sharing data. Open source analysis software is available for local use.	[131, 132]
Full Experimental Protocol with Data Analysis and Visualization	immunoSEQ® (Adaptive Biotechnologies)	Website, free account	DNA	No	TRB	Win, Mac, Linux	Web Browser	For analysis of Adaptive immunoSEQ data only	[133, 134]
	iPair (iRepertoire)	Free software download	Viable Cells	Only	TRA/TRB-, IgH/IgL-pairing	Win, Mac	Requires 64-bit OS	For analysis of data generated through iRepertoire's iPair service only	[135]
Data Analysis	RDI	Open-source	Aligned and mapped data (see notes)	Yes	TRA, TRB, TRG, TRD, IGL, IGK, IGH	Win, Mac, Linux	R v3.0.0+ with 4 additional packages	RDI calculates the Repertoire Dissimilarity Index; Uses aligned and mapped data from VDJpipe	[136, 137]
	TCRdist	Open-source	non-standard TSV file	Only	TRA, TRB	Mac, Linux	Python v2.7.11 with 5 additional packages	Attempts to describe generalizable, underlying features of epitope-specific repertoires	[138, 139]
	iRweb	Website (see notes)	Viable Cells	No	TRA, TRB, TRG, TRD, IGL, IGK, IGH	Win, Mac, Linux	Web Browser	A suite of bioinformatic tools, designed especially to work with iRepertoire's primer kits	[140]
	Loupe V(D)J Browser (10x Genomics)	Yes (see notes)	non-standard FASTQ-like format	Only	TRA/TRB-pairing	Linux, Win, Mac	Web Browser	This desktop application is used to analyze, search, and visualize V(D)J sequences and clonotypes from single cell data on the Chromium™	[141]
	tcR	Open-source	Aligned and mapped data (see notes)	Yes	TRA, TRB, TRG, TRD, IGL, IGK, IGH	Win, Mac, Linux	R v3.0.0 + with 14 additional packages	Accepts data that has already been aligned and mapped from MiTCR, MiGEC, VDJtools, immunoSEQ, IMSEQ or MiXCR; Available on CRAN	[142, 143]
	VDJtools	Open-source	Aligned and mapped data (see notes)	Yes	TRA, TRB, TRG, TRD, IGL, IGK, IGH	Win, Mac, Linux	Oracle Java 1.8, R v3.1.0+ with 14 additional packages	Accepts data that has already been aligned and mapped from MiTCR, MiGEC, IgBLAST (via MIGMAP wrapper), IMGT, immunoSEQ, VDJdb, Vidjil, RTCR, MiXCR, or ImSEQ	[144, 145]
	VDJviz	Open-source	Aligned and mapped data (see notes)	Yes	TRA, TRB, TRG, TRD, IGL, IGK, IGH	Win, Mac, Linux	Web Browser	Web-based GUI version of VDJtools	[146, 147]

The Notes column elaborates for cells indicating “see notes”

*CRAN* Comprehensive R Archive Network, *n/a* not applicable

### Next-Generation Immunosequencing Approaches

Next-generation sequencing (NGS) of the TCRβ and B cell receptor (BCR) IgH chain has enabled investigation of the adaptive immune repertoire from small blood or tissue samples. DNA/RNA from fluorescence-activated cell sorter-isolated immune cell subsets can be used to generate recombined receptor sequences using an Illumina sequencing platform [38•, 175••, 212, 213]. These technologies have quickly facilitated the generation of massive datasets (generally 5 × 10^4^–1 × 10^6^ sequence reads per sample) presented in proprietary and public databases (e.g., https://clients.adaptivebiotech.com/immuneaccess and for T1D sequences, http://clonesearch.jdrfnpod.org/). In turn, there is a need to develop new methods to parse, analyze, and graphically depict the extensive information [214]. Indeed, it is now possible to rapidly evaluate repertoire dynamics that include measures such as overlap across samples (Fig. 1a) and experimental groups, TCR sharing among multiple T cell subsets, comparisons of clonality and diversity, V(D)J gene usage (Fig. 1b), and public TCR sequences (Fig. 1c). Moreover, algorithms to identify individual clones by nucleotide and/or amino acid sequences, bioinformatics approaches are emerging to identify common peptide-binding motifs to facilitate the identification of classes of TCRs capable of binding shared peptides/epitopes [43].

High-depth NGS assays are limited by the current inability to simultaneously detect and pair TCRα and TCRβ sequences, yet these high-throughput discovery efforts provide a powerful clinical screening tool that retains the sensitivity to detect rare clones. Intermediate-depth applications such as RNA-seq or primer targeted sequencing yield less coverage and fewer sequence reads, but both TCR α- and β-chain sequencing can be obtained from laser-captured tissue microdissections or sorted lymphocyte subsets. Isolated RNA can be reverse transcribed to cDNA and amplified for transcriptome profiling to identify TCRs derived from alternate open reading frame transcripts and specific allelic expression [215]. Though moderate depth NGS does not allow for complete reconstruction of the TCR or identify exact epitope reactivity, these techniques can be used to characterize clonality, diversity, and gene segment usage for both the *TRA* and *TRB* genes. Adaptive pairSEQ allows for pairing of specific TCRα and TCRβ sequences by leveraging fractioning and probability based on relative abundance. However, there is a large input requirement and only highly prevalent sequences can be accurately paired.

Single-cell NGS tools involving directed-sequencing provide the ability to reconstruct the complete TCR with α- and β-chain pairing, which is required to confirm precise antigen reactivity and perform functional studies in vitro. Specifically, efforts that combine directed TCR α- and β-chain pairing with the additional analysis of lineage specific transcription factors and effector molecules not only provide the greatest opportunity for biomarker discovery but also have a bias toward detection of the most frequent clones [216]. Indeed, single-cell transcriptional profiling technologies offer high specificity and depth of information but low depth of coverage at considerable single-cell cost [175••]. New platforms and approaches are addressing this limitation, with droplet-based mRNA-seq or α- and β-chain sequence pairing potentially facilitating analysis of single cells with improved throughput capacity [43, 217••].

## TCRs as Biomarkers

### Limitations of Current T Cell Biomarkers

Marrero et al. identified islet antigen-reactive TRBV13-2 (Vβ8.2) public clones from the pLN of NOD mice. Importantly, a tolerogenic vaccine composed of an immunodominant peptide that binds the TCR Vβ8.2 chain prevented T1D in NOD mice [47] supporting the notion that public TCRs may serve as biomarkers and potential therapeutic targets. Currently, autoantibodies represent the best available biomarker to predict human T1D onset. At least 85% of children with multiple β-cell-reactive autoantibodies will progress to symptomatic disease within 15 years—with a lifetime risk approaching 100% [170•, 218]. Hence, there is a need to identify autoreactive T cell biomarkers in the pre-diabetic period, ideally prior to the loss of endogenous β-cell mass/function (Fig. 2). When combined with genetic risk markers and/or seropositivity for islet autoantibodies, T cell biomarker(s) may represent an early predictive index of disease; the goal, to employ these early clonal sequences as biomarkers of T1D progression, identify suitable candidates for therapy, develop novel T cell-targeted therapies, and monitor therapeutic responses.

Prior studies to assess cellular immunity and identify T cell biomarkers of T1D relied on immunoblot, T cell proliferation, ELISpot, and multimer staining with flow cytometry assays. These approaches can discriminate patients with T1D from healthy controls, though sensitivity and specificity are lower than assays detecting autoantibodies [15]. However, immunoblot and T cell proliferation assays often require freshly isolated cells, which limit their use in interventional trial settings [219]. Conversely, ELISpot and MHC multimer-based screening methods are able to precisely identify the target antigen and epitope as well as T cell phenotype, but sample requirements and HLA restriction hinder multi-center utility. Through TCR and transcriptional profiling, investigators now have the capacity to identify T cell clones, phenotype, and the specific epitope reactivities of T cells regardless of HLA haplotypes.

### Lessons From Studies of Infectious Disease and Cancer

As a well-studied and penetrant pathogen in the US general population, cytomegalovirus (CMV) provided a useful model for studies of TCR β-chain sequencing [166••]. Through high-throughput analyses coupled with machine learning, investigators were able to identify CMV-reactive TCRs and accurately infer CMV seropositivity status from the TCR repertoire with area under the receiver operating characteristic curve (AUROC) = 0.94 (Fig. 1d). Interestingly, only 164 of the 488 (34%) CMV-reactive TCRβ identified were significantly associated with CMV seropositivity [166••]. Perhaps not surprisingly, the authors noted that public CMV-associated TCRβ sequences were only identified from HLA-matched cohorts, supporting the notion that TCR biomarkers are HLA restricted. Altogether, these findings suggest that given a sufficiently large cohort, it may be possible to build computer algorithms to determine an individual’s likelihood of progression to T1D based upon TCR repertoire analysis, for specific HLA types.

Similar efforts in the field of cancer research have led to advancements in treatment and monitoring of disease. Notably, immune receptor deep sequencing can now be used to characterize acute lymphoblastic leukemia as monoclonal or polyclonal, identify the malignant T cell or B cell developmental stage, monitor disease progression, and track therapeutic response [220•]. NGS is also of great value for monitoring rare T cell lymphomas, which are difficult to detect by traditional methods and consequently, are associated with poor prognosis [221]. When sequencing is performed at baseline prior to treatment, the TCR or BCR repertoire can be followed throughout therapy, and minimal residual disease (MRD), which is considered the most reliable means to predict patient outcome and potential for relapse, can be evaluated with greater sensitivity via immunosequencing (i.e., 1 in 10^6^ cells) compared to previous standard methodologies (i.e., flow cytometry or allele-specific oligonucleotide PCR (ASO-PCR)) [220•, 222, 223]. These concepts have the potential to be translated to T1D research, wherein autoreactive clones could be monitored to evaluate therapeutic response following immunomodulatory therapies, with the goal to detect persistent autoreactive memory lymphocytes in circulation and potentially track relapsing and remitting phases characteristic of many autoimmune diseases [224].

### Novel Methodology to Improve Detection

When considering how to apply TCR biomarkers in T1D clinical trials, tissue restriction of the T cell repertoire represents a critical limitation [38•]. Indeed, our recent study suggested that CD4^+^ T cell clones are highly tissue restricted while CD8^+^ T cells clones may be present in multiple tissues and in circulation (Fig. 1a) [38•]. Because T cells targeting β-cell antigens are most abundant within the pLN or the pancreas itself [38•], strategies are needed to improve recovery of autoreactive clones from accessible tissues (i.e., peripheral blood). To address this, Thelin et al. utilized subcutaneous scaffolds bearing β-cell lysates to draw an enriched population of autoreactive T cells from the circulation of NOD mice [225]. While further investigation is clearly needed, a safe analogous methodology could be utilized to improve the recovery of autoreactive T cell clones following a local challenge. Beyond this, longitudinal studies are needed to identify the initiating autoreactive TCRs, address whether there is evidence of epitope spreading at the clonal level, and to characterize antigenic spreading throughout the pre-T1D period (Fig. 2).

### TCR Sequencing in T1D

Efforts to identify public TCR using NGS are still in the early stages in the field of T1D research. Thus far, the greatest challenge stems from high repertoire diversity and low levels of clonal overlap, when comparing across subjects and tissues. In our study of 18 T1D and nine control subjects, deep sequencing of TCRs from T cell subsets (isolated from pLN, spleen, PBMC, and inguinal or mesenteric LN) showed that limited Treg or conventional CD4^+^ T cell (Tconv) repertoire overlap exists across tissues and in the circulation within a given individual (i.e., 3–4% overlap between pLN and PBMC for T1D donors) [38•]. Comparatively, within the CD8^+^ T cell subset, approximately 20% of clones were shared across pLN and PBMC [38•]. Further, for one donor, we were able to sequence the intra-islet T cell isolate. We found only seven CD4^+^ TCR clones conserved across islet and pLN samples. However, 58 CD8^+^ TCRs were detected from both pLN and islet tissues. Within this cohort, seven HLA-A*02 matched T1D donors were examined for public TCRs within the pLN and 14 unique CDR3 sequences were common across the CD8^+^ population from each of the seven donors [38•]. Hence, we expect that CD8^+^ T cells may serve as a more reliable T cell biomarker in T1D.

The sheer number of unique sequences present has raised a new challenge as investigators search for public autoreactive clone(s), a so-called needle in a haystack. In fact, contrary to our original hypothesis, global TCR repertoire analysis could not be used to discriminate T1D from control donors by broad *TRB* gene usage. However, when we queried TCR NGS data for known autoreactive T cell clonotypes, we identified 59 unique CDR3 β-chain amino acid sequences, the majority of which were more prevalent in T1D donor tissues versus control or type 2 diabetes samples [38•]. Moreover, we reported the CDR3 β-chain sequence of the GAD-reactive TCR clone (4.13) to be highly enriched for seven T1D donors and one autoantibody negative control subject with T1D permissive HLA, perhaps representing a key driver of T1D pathogenesis in these subjects. However, studies of multiple T cell subsets, autoantibody positive organ donors, and longitudinal studies of living subjects are needed to further characterize the pathogenic or protective role of this clone.

As noted above, Han et al. observed common sequence motifs, where nucleic acid alterations within the V(D)J junction resulted in TCRs with variability at the amino acid level but similar tumor antigen reactivities [43]. This notion of shared motifs must be explored further in T1D where a particular public clone seems unlikely to be present in all or even the majority of patients, but pathogenic T cells with common antigen reactivities are expected. Together, these observations point toward a need for machine learning algorithms to determine conserved autoreactive TCR repertoires concurrent with established biomarkers (i.e., autoantibodies, genetic risk alleles), early in the disease process when intervention is most likely to impact disease course.

## TCRs as Therapeutics

### Clinical Response to T Cell Targeting Therapeutics

For nearly 50 years, immunomodulatory agents, including those aimed at T cell blockade, have been prescribed to prevent rejection and graft versus host disease in solid organ and bone marrow transplant recipients [226–230]. Immunosuppressants targeting global or precise T cell frequency and function have since been applied for the treatment of autoimmune diseases, such as multiple sclerosis (MS) and rheumatoid arthritis (reviewed in [231, 232]). T cell-targeted therapies have been tested in multiple T1D clinical trials aiming to interdict in the destruction of β-cells and preserve C-peptide production [233–249]. Though no T cell-targeted therapy has achieved long-term remission with clinical equipoise, evidence of temporary or partial efficacy in subsets of responder subjects has been observed following treatment with antithymocyte globulin (ATG) alone or in combination with granulocyte colony stimulating factor (G-CSF), teplizumab, alefacept, or abatacept [236, 242, 243, 246, 247, 249, 250].

ATG, cyclophosphamide, and G-CSF were key components of autologous non-myeloablative hematopoietic stem cell transplants, which restored insulin independence in patients with T1D but conferred high risk of morbidity (e.g., severe neutropenia requiring extended hospitalization, gonadal dysfunction, alopecia) [251, 252]. ATG induces T cell apoptosis and complement-dependent lysis, B cell apoptosis, modulation of antigen presenting cell surface molecules and induction of Tregs [253] while G-CSF stimulates hematopoietic mobilization [254]. Due to the high-risk nature of this approach, several groups have attempted to deconstruct this combination approach in hopes of designing lower risk therapies that maintain efficacy. Specifically, Haller et al. recently demonstrated that low-dose ATG plus G-CSF preserved C-peptide for 12 months in subjects with established T1D (4–24 months post-diagnosis), with responders characterized by older age at disease onset [246]. Combination treatment with low dose ATG and G-CSF induced significant and sustained immunomodulation including elevated Tregs, reduced CD4^+^ and increased CD8^+^ T cell frequency, increased CD16^+^CD56^hi^ NK cell frequency, and increased frequency of CD4^+^PD-1^+^ T_CM_ [247]. Accordingly, NGS studies in our lab are ongoing to evaluate precise effects of therapy on the TCR repertoire. Earlier intervention is expected to provide improved efficacy, and a study of ATG plus G-CSF combination therapy in new-onset T1D subjects will reach the primary endpoint in late 2017 (NCT02215200).

Development of humanized monoclonal antibodies and immunomodulatory fusion proteins have led to similar trials of T cell-directed therapies—namely, anti-CD3 (teplizumab), LFA-3/Fc fusion protein (alefacept), and CTLA-4/Fc fusion protein (abatacept). While space limitations preclude an extensive review of these studies, it is important to note that each of these agents has demonstrated signal indicating capacity to preserve C-peptide through T cell modulation. That said, it is not yet known how these T cell modulating agents (i.e., anti-CD3, alefacept, abatacept, or ATG ± G-CSF) impact the autoreactive T cell repertoire, and detailed investigations within prevention and reversal trials are warranted. We speculate that early intervention, prior to epitope spreading, may provide the best opportunity to deplete immunodominant autoreactive clonotypes using monoclonal antibodies or similar agents (Fig. 2). Moreover, selection of the optimal biologic for tailored immune therapy may depend upon subject age, T1D disease stage, or additional biomarkers, which have yet to be defined. Identification of autoreactive T cell clone(s) through NGS could provide candidate targets for monoclonal therapy, ideally, before autoantibody development occurs. These notions support ongoing investigation to identify key pathogenic clones as targets for T cell-directed immunotherapy.

### The Engineered TCR and CAR-T Experience

Autologous chimeric antigen receptor (CAR) T cells, engineered to express a tumor antigen-specific immunoglobulin variable region (single-chain variable fragment (scFv)) fused to TCR and costimulatory signaling domains, represent a promising therapeutic strategy for the treatment of solid tumors, B cell leukemia, and lymphoma [255–258]. A particular benefit in settings of cancer lies in the ability to deplete malignant cells without the requirement for antigen presentation via HLA; likewise, lack of HLA restriction could enable non-autologous T cell sources. Indeed, CAR-Tregs could be generated for application in T1D by expressing a β-cell antigen-reactive immunoglobulin scFv providing tissue-targeted activation and eliciting bystander suppression. However, limitations exist related to the requirement for surface expression of the target antigen(s) on islets or β-cells. Alternatively, TCR gene transfer facilitates targeted Treg therapies that recognize intra-cellular β-cell antigens presented by HLA following native antigen uptake and presentation via antigen presenting cells. TCR gene transfer can be accomplished via mRNA electroporation for transient expression [259, 260] or lentiviral transduction for stable TCR expression [261]. The former approach limits risk by bypassing the need for vector integration, but there may be a requirement for repeat dosing due to transient expression. Whereas in settings of T1D, longer-lived TCR “avatars” generated through lentiviral transduction may induce long-term bystander suppression and, potentially, lead to infectious tolerance to multiple autoantigens ([262] and Yeh et al., in press).

Through detailed studies using experimental autoimmune encephalomyelitis (EAE) murine models of MS, investigators have elucidated the timeline for epitope spreading throughout disease pathogenesis, indicating the optimal time points to initiate tolerogenic cell therapy [263–265]. Longitudinal investigations of epitope spreading in human T1D patients are needed and may be best accomplished through TCR repertoire profiling of characterized receptors. Such efforts could be used to outline tailored therapies involving engineered Treg avatars specific for identified autoantigen(s) (e.g., insulin, GAD, IA-2, HIPs, or insulin DRiP peptides, described above). Given that the argument for equipoise is undoubtedly different in settings of T1D versus hematologic malignancy, there is a need to validate Treg avatar lineage stability and introduce additional safety mechanisms, such as suicide genes [266]. With these notions in mind, a more complete understanding of the TCR repertoire may identify public Treg TCRs and/or antigen-reactivities that are particularly poised to induce tolerance in T1D.

## Moving Forward

The emergence of high-throughput immunosequencing and improved access to pathogenic lymphocytes within the target organ and draining LN have enabled searching for T cell biomarkers of T1D. T cell repertoire characterization of human pancreas samples within the nPOD bioresource bank will serve as a training set for moving TCR biomarkers into the clinical space. Deep sequencing of TCRs is expected to provide the field with sensitive tools to predict disease and monitor therapeutic responses. While the search for T1D-initiating TCRs as biomarkers may not be successful (i.e., every patient’s path to autoimmunity may be unique), knowledge of immunosequences/motifs could nonetheless aid in tracking immune responses within an individual and could potentially be used to generate antigen-specific Tregs for adoptive cell therapy. TCR-specific treatments may ultimately yield durable therapeutic benefit beyond the transient effects seen with current, non-specific T cell-directed therapies, alone or in combination with tolerance induction strategies. Beyond this, the capacity to monitor and detect expansions of rare but long-lived immune memory presents the potential to monitor patients over time and pre-emptively redose and block autoimmune recurrence following restorative therapies.

Limited access to the pancreas in living subjects will likely represent a key challenge as we attempt to detect from PBMC the autoreactive clones originally identified from deceased organ donor tissues (i.e., pancreas, spleen, LN) [38•]. Longitudinal studies including birth cohort samples are needed to determine key pathogenic T cell clones or autoreactive motifs present in the earliest disease stages, potentially prior to insulitis or even autoantibody development. We expect that the vast collection of data and knowledge stemming from immunosequencing efforts will markedly improve our understanding of the immunopathogenesis of T1D and bring us closer to our ultimate goals of preventing and reversing T1D.
